# Relation of insulin treatment for type 2 diabetes to the risk of major adverse cardiovascular events after acute coronary syndrome: an analysis of the BETonMACE randomized clinical trial

**DOI:** 10.1186/s12933-021-01311-9

**Published:** 2021-06-22

**Authors:** Gregory G. Schwartz, Stephen J. Nicholls, Peter P. Toth, Michael Sweeney, Christopher Halliday, Jan O. Johansson, Norman C. W. Wong, Ewelina Kulikowski, Kamyar Kalantar-Zadeh, Henry N. Ginsberg, Kausik K. Ray

**Affiliations:** 1 Division of Cardiology, University of Colorado School of Medicine, 1700 N. Wheeling St. (Cardiology 111B), Aurora, CO 80045 USA; 2 Victorian Heart Institute, Monash University, Melbourne, Australia; 3 Cicarrone Center for the Prevention of Cardiovascular Disease, Johns Hopkins University School of Medicine, Baltimore, MD USA; 4 CGH Medical Center Sterling, Sterling, IL USA; 5 Resverlogix Corporation, Calgary, AB Canada; 6 Division of Nephrology and Hypertension, University of California Irvine, Orange, CA USA; 7 Department of Medicine, Vagelos College of Physicians and Surgeons, Columbia University, New York, NY USA; 8 Imperial Centre for Cardiovascular Disease Prevention, Imperial College, London, UK

**Keywords:** Acute coronary syndrome, Diabetes, Epigenetics, BET proteins

## Abstract

**Background:**

In stable patients with type 2 diabetes (T2D), insulin treatment is associated with elevated risk for major adverse cardiovascular events (MACE). Patients with acute coronary syndrome (ACS) and T2D are at particularly high risk for recurrent MACE despite evidence-based therapies. It is uncertain to what extent this risk is further magnified in patients with recent ACS who are treated with insulin. We examined the relationship of insulin use to risk of MACE and modification of that risk by apabetalone, a bromodomain and extra-terminal (BET) protein inhibitor.

**Methods:**

The analysis utilized data from the BETonMACE phase 3 trial that compared apabetalone to placebo in patients with T2D, low HDL cholesterol, andACS. The primary MACE outcome (cardiovascular death, myocardial infarction, or stroke) was examined according to insulin treatment and assigned study treatment. Multivariable Cox regression was used to determine whether insulin use was independently associated with the risk of MACE.

**Results:**

Among 2418 patients followed for median 26.5 months, 829 (34.2%) were treated with insulin. Despite high utilization of evidence-based treatments including coronary revascularization, intensive statin treatment, and dual antiplatelet therapy, the 3-year incidence of MACE in the placebo group was elevated among insulin-treated patients (20.4%) compared to those not-treated with insulin (12.8%, P = 0.0001). Insulin treatment remained strongly associated with the risk of MACE (HR 2.10, 95% CI 1.42–3.10, P = 0.0002) after adjustment for demographic, clinical, and treatment variables. Apabetalone had a consistent, favorable effect on MACE in insulin-treated and not insulin-treated patients.

**Conclusion:**

Insulin-treated patients with T2D, low HDL cholesterol, and ACS are at high risk for recurrent MACE despite the use of evidence-based, contemporary therapies. A strong association of insulin treatment with risk of MACE persists after adjustment for other characteristics associated with MACE. There is unmet need for additional treatments to mitigate this risk.

*Trial registration* ClinicalTrials.gov NCT02586155, registered October 26, 2015

## Introduction

Patients with acute coronary syndrome (ACS) are at high risk for additional major adverse cardiovascular events (MACE). Patients with type 2 diabetes comprise approximately 30% of those with ACS and experience up to twice the risk of recurrent MACE as those without type 2 diabetes [1–3].

Chronic insulin treatment is required in approximately 25% of patients with type 2 diabetes to control hyperglycemia [4]. Among patients with type 2 diabetes and ACS, approximately 35% receive chronic insulin treatment [5–7]. In patients with type 2 diabetes who have not had prior MACE or who have stable coronary heart disease, insulin use is associated with elevated risk of incident or recurrent MACE and death despite use of evidence-based cardiovascular therapies [8–11]. However, the extent to which insulin treatment is associated with elevated cardiovascular risk after ACS is uncertain. Moreover, no diabetes medication has been shown to reduce MACE after ACS. Medications that have shown favorable cardiovascular effects in stable patients have not been studied after ACS [metformin, sodium–glucose loop transporter-2 (SGLT2) inhibitors] and drugs in other classes that have been studied after ACS failed to show benefit [glucagon-like peptide-1 (GLP-1) receptor agonists, dipeptidyl peptidase-4 inhibitors] [6, 12].

Bromodomain and extra-terminal (BET) proteins are epigenetic regulators of gene transcription. Apabetalone is a selective BET protein inhibitor with potentially salutary effects on pathways implicated in inflammation, endothelial dysfunction, thrombosis, and vascular calcification [13–15]. These injurious processes are prognostically important in patients with ACS [16]. Phase 2 clinical data suggested that apabetalone might have favorable effects on MACE, particularly among those with type 2 diabetes [17]. Accordingly, the phase 3 BETonMACE trial [7] was designed to compare apabetalone with placebo in patients with type 2 diabetes and recent ACS. The present analysis used data from BETonMACE to determine the association of insulin use with risk of MACE after ACS in a contemporary cohort of patients receiving evidence-based background cardiovascular treatments. In addition, we evaluated the interaction of insulin use and apabetalone on that risk.

## Methods

### Study design

The design and principal results of the BETonMACE trial have been described [7, 18]. The study was approved by the responsible institutional review board at each participating site, and each patient gave written, informed consent. In brief, inclusion criteria were age at least 18 years, ACS within the preceding 7–90 days, low high-density lipoprotein cholesterol (HDL-C) levels, and a diagnosis of type 2 diabetes. Concomitant high-intensity statin therapy with atorvastatin 40–80 mg or rosuvastatin 20–40 mg daily was required unless a lower dose was medically indicated. Other concomitant treatments, including therapies for type 2 diabetes, were assessed at the randomization and subsequent visits. Patients with a prescription for any insulin product were considered to be insulin-treated. Eligible patients were randomized to treatment with apabetalone 100 mg twice daily or matching placebo. The trial continued until a blinded clinical events committee determined that at least 250 patients had experienced the primary MACE outcome (cardiovascular death, non-fatal myocardial infarction or non-fatal stroke). Secondary endpoints included hospitalization for heart failure. Adverse and serious adverse events, including hypoglycemia, were reported by investigators and categorized according to the Medical Dictionary for Regulatory Activities (MedDRA).

### Statistical analysis

This was a post hoc analysis. Baseline characteristics were summarized as percentages for dichotomous data and means (SDs) for approximately normal or medians (IQRs) for non-normal continuous data. Characteristics were compared between patients with diabetes who were or were not treated with insulin during the trial using t-tests or Wilcoxson tests for continuous variables and chi-square tests for categorical variables.

The cumulative incidence of the primary MACE endpoint was described in each treatment group and insulin use subgroup with Kaplan–Meier survival analysis. Cox proportional hazards models were used to determine treatment hazard ratios (HR) with 95% confidence interval (CI) and interaction of study treatment and insulin treatment on MACE.

Cox regression models were used to determine whether insulin treatment was an independent predictor of MACE and hospitalization for heart failure in the placebo group. Model 1 was stratified for country and baseline statin allocation. Model 2 was adjusted for demographic variables (age, sex, race) and other characteristics that differed between insulin-treated and not insulin-treated patients including duration of diabetes; history of heart failure, myocardial infarction, or coronary revascularization procedure prior to the qualifying ACS; statin treatment intensity, and hemoglobin A1c. Model 3 was also adjusted for the variables in model 2 plus use of metformin, sulfonylureas, sodium–glucose cotransporter-2 (SGLT2) inhibitors, and glucagon-like peptide-1 (GLP-1) receptor agonists.

Heterogeneity in the absolute difference in risk of MACE with apabetalone versus placebo according to insulin treatment category was determined according to equation 5 for quantitative interaction in the treatise of Gail and Simon [19]. The occurrence of adverse events related to hypoglycemia in insulin-treated versus not insulin-treated patients and of MACE among those with or without hypoglycemia were compared with Fisher’s exact test. Other analyses were performed with R software, version 3.5.1 or higher (R Foundation for Statistical Computing). P-values less than 0.05 were considered statistically significant.

## Results

The analysis cohort comprised 2418 patients who were randomized at 190 sites in 13 countries between November 2015 and July 2018, received at least one dose of study medication, and were followed for a median of 26.5 months. Overall, there were 274 primary MACE endpoints with 125 (10.9%) in the apabetalone group and 149 (12.4%) in the placebo group (treatment HR 0.82; 95% CI 0.65–1.04; P = 0.11) [7]. There were 78 patients with hospitalization for heart failure, with 29 (2.4%) in the apabetalone group and 49 (4.0%) in the placebo group (treatment HR 0.59; 95% CI 0.38–0.94; P = 0.03) [20].

Baseline characteristics of the analysis cohort are summarized in Table 1. There were 829 (34.3%) insulin-treated patients and 1589 (65.7%) who were not insulin-treated. Insulin-treated patients were more likely to be female, of non-white race, to have a longer duration of diabetes and a history of heart failure, to have a prior history of myocardial infarction or coronary revascularization, and to receive high-intensity statin treatment. Insulin-treated patients had higher baseline levels of fasting glucose and hemoglobin A1c than patients not treated with insulin. Insulin-treated patients were less likely to be treated with metformin or sulfonylureas, but more likely to be treated with SGLT2 inhibitors or GLP-1 receptor agonists.

**Table 1:** Baseline characteristics of the patients according to insulin treatment

	All patients (N = 2418)	Insulin-treated (N = 829)	Not insulin-treated (N = 1589)	Treated vs. not treated P-value
Demographics				
Age, years, mean (SD)	61.3 (9.5)	61.0 (9.4)	61.4 (9.6)	0.36
Female, n (%)	618 (25.6)	239 (28.8)	379 (23.9)	0.008
Non-White Race, n (%)	299 (12.4)	140 (16.9)	159 (10.0)	< 0.0001
Medical history				
Duration of diabetes, years, mean (SD)	8.5 (7.6)	12.6 (8.0)	6.4 (6.5)	< 0.0001
Prior MI, PCI, or CABG; n (%)	865 (35.8)	331 (39.9)	534 (33.6)	0.002
Heart failure; n (%)	348 (14.4)	141 (25.0)	207 (13.0)	0.01
Index ACS, n (%)				
STEMI	932 (52.7)	313 (37.8)	619 (39.0)	0.24
Non-STEMI	836 (47.3)	304 (36.7)	532 (33.5)	0.24
Unstable angina	625 (26.0)	200 (24.1)	425 (26.7)	0.19
Revascularization for index ACS	1922 (79.5)	667 (80.5)	1255 (79.0)	0.42
Biometrics, mean (SD)				
Body mass index, kg/m^2^	30.3 (4.9)	30.2 (4.9)	30.3 (4.9)	0.66
Systolic blood pressure, mmHg	129 (15)	130 (16)	129 (14)	0.24
Cardiovascular and diabetes medications, n (%)				
High-intensity statin	2195 (90.2)	770 (92.9)	1425 (89.7)	0.01
ACE-inhibitor or ARB	2229 (92.2)	770 (92.9)	1459 (91.8)	0.40
Dual anti-platelet therapy	2122 (87.8)	735 (88.7)	1387 (87.3)	0.36
Metformin	1998 (82.6)	604 (72.9)	1394 (87.7)	< 0.0001
Sulfonylurea	707 (29.2)	177 (21.4)	530 (33.4)	< 0.0001
SGLT2 inhibitor	298 (12.3)	137 (16.5)	161 (10.1)	< 0.0001
GLP-1 receptor agonist	86 (3.6)	51 (6.2)	35 (2.2)	< 0.0001
Clinical chemistry, median (Q1–Q3)				
Estimated GFR (mL/min/1.73m^2^)	98.3 (76.2–126.2)	95.7 (73.8–127.9)	99.7 (77.3–125.5)	0.23
Fasting glucose, mmol/L	7.5 (6.1–9.7)	8.7 (6.8–11.4)	7.0 (5.9–9.0)	< 0.0001
Hemoglobin A1c, %	7.3 (6.4–8.7)	8.4 (7.5–9.6)	6.9 (6.2–7.8)	< 0.0001
LDL cholesterol, mmol/L	1.7 (1.3–2.2)	1.7 (1.3–2.2)	1.7 (1.3–2.2)	0.62

**Fig. 1:**
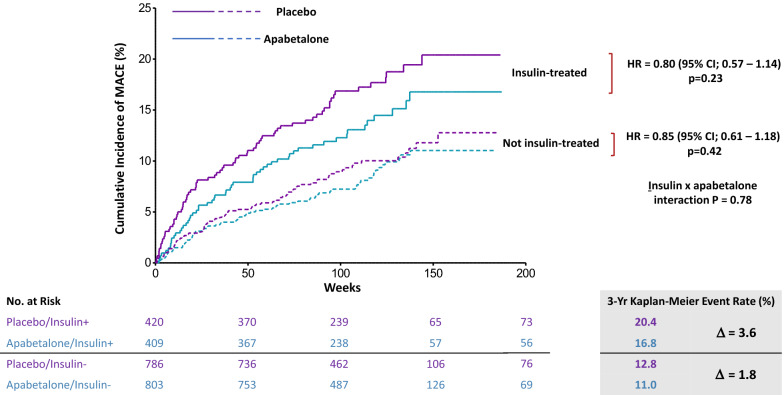
Cumulative incidence of MACE by study treatment group and insulin treatment category. Kaplan–Meier plots showing the cumulative incidence of MACE in the apabetalone and placebo groups, according to insulin treatment category. Hazard ratio are calculated by Cox proportional hazards models, stratified by country (countries with fewer than 100 patients combined) and statin agent (atorvastatin or rosuvastatin). Solid lines, insulin-treated. Dashed lines, not insulin-treated. MACE, major adverse cardiovascular events. Apabetalone had a larger effect on absolute risk of MACE among insulin-treated than not insulin-treated patients (quantitative interaction P = 0.006)

The Fig. 1 shows the cumulative incidence of MACE by insulin treatment category and study treatment group. Among patients assigned to placebo, Kaplan–Meier estimates of the incidence of MACE at 3 years were 20.4% in the insulin-treated subgroup and 12.8% in the not insulin-treated subgroup (relative risk 1.59; P = 0.0001). Apabetalone had a similar effect on the relative risk of MACE in the insulin-treated subgroup (treatment HR 0.80; 95% CI 0.57–1.14) and in the not insulin-treated subgroup (HR 0.85; 95% CI 0.61–1.18; P_interaction_ = 0.78). However, due to the higher absolute risk of MACE in the former subgroup, the 3-year Kaplan–Meier estimate of the absolute difference in risk of MACE between apabetalone and placebo groups was twice as great (3.6%) as in the not insulin-treated subgroup (1.8%; P = 0.006 for heterogeneity).

Table 2 shows the results of Cox proportional hazards models relating insulin treatment to the risk of MACE and hospitalization for heart failure in the placebo group. Insulin use was a significant predictor of MACE in unadjusted and adjusted models. In fully adjusted Model 3 the use of insulin was associated with a HR for MACE of 2.10 (95% CI 1.42–3.10; P = 0.0002). Insulin use was also a significant predictor of hospitalization for heart failure with HR 2.34 (95% CI 1.19–4.60; P = 0.01) in the fully adjusted model.

**Table 2:** Cox proportional hazards models for the association of insulin treatment with risk of MACE and hospitalization for heart failure in the placebo group

Insulin-treated (n=420)	Not insulin treated (n=786)	Model	Model covariates	HR [95% CI] (insulin-treated/not treated)	p-value
No. of events/N (%)	No. of events/N (%)				
MACE 73/420 (17.4) HHF 28/420 (6.7)	MACE 76/786 (9.7) HHF 21/786 (2.7)	1	Unadjusted	MACE	
				1.89 [1.36–2.62]	0.0001
				HHF	
				2.48 [1.40–4.40]	0.002
		2	Age, sex, race, duration of diabetes, HbA1c, use of intensive statin, prior MI/PCI/CABG, and prior heart failure	MACE	
				1.86 [1.27–2.73]	0.002
				HHF	
				1.79 [0.92–3.47]	0.08
		3	Model 2 plus adjustment for use of metformin, sulfonylurea, SGLT2i, and GLP-1 RA	MACE	
				2.10 [1.42–3.10]	0.0002
				HHF	
				2.34 [1.19–4.60]	0.01

*CABG* coronary artery bypass grafting, *GLP-1 RA* glucagon-like peptide-1 receptor agonist, *HHF* hospitalization for heart failure, *MACE* major adverse cardiovascular events, *MI* myocardial infarction, *PCI* percutaneous coronary intervention, *SGLT2i* sodium–glucose loop transporter 2 inhibitor

The incidence of treatment-emergent adverse events (TEAE) (i.e., adverse events that developed or worsened during randomized treatment) was greater in patients treated with insulin than in those not treated with insulin (79% versus 62%), with the difference driven by cardiovascular (28% versus 20%) and hematologic (7% versus 3%) events. Similarly, serious TEAE (those that were fatal, life-threatening, required or prolonged hospitalization, or led to disability) occurred in 39% of insulin-treated versus 23% of not insulin-treated patients. TEAE rates were similar in apabetalone versus placebo groups among patients treated with insulin (77% versus 82%) or not treated with insulin (64% versus 61%) as were serious TEAE rates.

Adverse events related to hypoglycemia were more common in insulin-treated patients (n = 26, 3.1%) than in patients not treated with insulin (n = 11, 0.7%, P < 0.001). There was 1 serious adverse event related to hypoglycemia, in an insulin-treated patient. The number of patients with a hypoglycemia-related adverse event was similar with apabetalone or placebo (17 vs. 20). Hypoglycemia did not appear to account for the increased risk of MACE in insulin-treated patients. Among the 26 insulin-treated patients with a hypoglycemia-related adverse event, MACE occurred in 5 (19.2%). Among the 803 insulin-treated patients without a hypoglycemia-related adverse event, MACE occurred in 124 (15.4%, P = 0.58).

## Discussion

Insulin treatment has been associated with elevated risk for incident or recurrent MACE in patients with type 2 diabetes without or with established, stable atherosclerotic cardiovascular disease [8–11]. Patients with ACS are at high risk of further ischemic cardiovascular events. This risk is magnified approximately twofold among patients with type 2 diabetes [1–3]. The present analysis demonstrates that risk is even further magnified in insulin-treated patients with type 2 diabetes and ACS, with a 3-year incidence of cardiovascular death, myocardial infarction, or stroke of approximately 20%. This elevated risk persists despite high utilization of contemporary, evidence-based treatments for ACS including coronary revascularization, intensive statin therapy, inhibitors of the renin-angiotensin system, and dual anti-platelet agents and with good control of blood pressure and low-density lipoprotein cholesterol levels. Insulin use was also associated with a significantly increased risk of hospitalization for heart failure, even after adjustment for prior heart failure.

Insulin treatment was associated with other characteristics generally associated with high cardiovascular risk, including prior myocardial infarction, coronary revascularization, and heart failure and longer duration of diabetes. In particular, a longer duration of diabetes may be associated with more severe or extensive atherosclerosis [21–23]. Insulin use was also associated with higher levels of hemoglobin A1c and fasting glucose and lower utilization of metformin and sulfonylureas. On the other hand, insulin use was also associated with greater utilization of SGLT2 inhibitors and GLP-1 receptor agonists which might be expected to reduce risk of recurrent MACE and heart failure events. However, adjustment for all of these variables and demographic characteristics did not diminish the strength of association of insulin treatment with risk of MACE and hospitalization for heart failure. We cannot exclude the possibility that other, unidentified covariates account in part for the remaining association.

Insulin may also have direct adverse cardiovascular effects. Exogenous insulin therapy may cause hypoglycemia and in turn increased risk of MACE and death [24–26]. Insulin also promotes pathologic cell growth and proliferation in the arterial wall [27]. Intravascular ultrasound shows that insulin-treated patients with coronary artery disease have smaller external elastic membrane and lumen volumes than patients not treated with insulin, resulting in greater percent atheroma volume for a given total atheroma volume [28]. In a favorable direction, insulin treatment reduces the concentration of circulating free fatty acids [29] and thereby might attenuate deleterious effects of elevated free fatty acids on endothelial function, intensity of inflammation, blood pressure, and sudden cardiac death [30, 31].

The only randomized, prospective trial evaluating effects of long-term insulin treatment on risk of MACE following ACS in patients with type 2 diabetes indicated no benefit and the possibility of harm. The Diabetes Mellitus Insulin Glucose Infusion in Acute Myocardial Infarction 2 (DIGAMI-2) trial comprised 1253 patients of whom 947 were randomly assigned to insulin-based or conventional glucose control at hospital discharge after receiving 24 h of insulin–glucose infusion in hospital. Two year mortality was high but did not differ significantly between groups (23.4% versus 21.2%) [32]. In extended follow-up at a median 4.1 years, insulin-based treatment was also associated with a non-significant excess of deaths (odds ratio 1.30, 95% CI 0.93–1.81) and with a significant excess of non-fatal MACE (odds ratio 1.89, 95% CI 1.35–2.63) [33]. Other diabetes drugs evaluated in large randomized, placebo-controlled trials in patients with ACS also failed to show benefit, including lixisenatide [6], alogliptin [12], and aleglitazar [5].

### Limitations

Limitations include the fact that the analysis was conducted on a post hoc basis and the trial had limited power to draw inference on event rates and treatment effects in subgroups. Cox models were adjusted for characteristics that differed between patients who were or were not treated with insulin. However, the possibility of residual confounding by unmeasured clinical or laboratory variables cannot be excluded. Changes in the doses of diabetes medications were not ascertained. Only 3.1% of insulin-treated patients had an investigator-reported adverse event related to hypoglycemia and these patients accounted for only 5 MACE events; however, some hypoglycemic events may not have been reported and may have contributed to a higher rate of MACE in insulin-treated patients. Patients in the BETonMACE trial were randomized 7–90 days after the qualifying ACS event; i.e., at hospital discharge or thereafter. The current analysis therefore does not consider potential effects of insulin use during hospitalization for ACS and associated revascularization procedures [34]. Patients in the trial had low levels of HDL cholesterol; it is uncertain whether findings were influenced by this selection criterion.

## Conclusion

Insulin treatment is required in many patients with type 2 diabetes and ACS. Notwithstanding the above limitations, insulin use is strongly associated with risks of recurrent MACE and hospitalization for heart failure following ACS, even after adjustment for other characteristics associated with these risks and despite widespread use of evidence-based therapies including coronary revascularization, intensive statin treatment, inhibitors of the renin–angiotensin system, and dual-antiplatelet agents. Because no diabetes drug has shown cardiovascular benefit in patients with recent ACS, the present observations highlight a need for new treatments to reduce MACE and heart failure events in this very high-risk setting.

Apabetalone is a selective BET protein inhibitor with potentially salutary effects on pathways implicated in inflammation, endothelial dysfunction, thrombosis, and vascular calcification [13–15]. In patients with type 2 diabetes, low HDL cholesterol, and recent ACS, the BETonMACE study showed a trend to decreased risk of MACE with apabetalone and fewer heart failure hospitalizations compared to placebo [7, 20]. The large absolute difference in the incidence of MACE with apabetalone versus placebo among insulin-treated patients suggests that this group might derive benefit and warrants further study.

## Data Availability

The authors declare that the data supporting the findings of this study are available within the article.

## Abbreviations

ACS: Acute coronary syndrome
BET: Bromodomain and extra-terminal domain
DIGAMI-2: Diabetes Mellitus Insulin Glucose Infusion in Acute Myocardial Infarction 2
GLP-1: Glucagon-like peptide-1
MACE: Major adverse cardiovascular events
SGLT2: Sodium–glucose cotransporter-2

## References

[1] Ahmed S, Cannon CP, Murphy SA, Braunwald E (2006). Acute coronary syndromes and diabetes: is intensive lipid lowering beneficial? Results of the PROVE IT-TIMI 22 trial. Eur Heart J.

[2] Ray KK, Colhoun HM, Szarek M, Baccara-Dinet M, Bhatt DL, Bittner VA (2019). Effects of alirocumab on cardiovascular and metabolic outcomes after acute coronary syndrome in patients with or without diabetes: a prespecified analysis of the ODYSSEY OUTCOMES randomised controlled trial. Lancet Diabetes Endocrinol.

[3] Schupke S, Neumann FJ, Menichelli M, Mayer K, Bernlochner I, Wohrle J (2019). Ticagrelor or prasugrel in patients with acute coronary syndromes. N Engl J Med.

[4] Wallia A, Molitch ME (2014). Insulin therapy for type 2 diabetes mellitus. JAMA.

[5] Lincoff AM, Tardif JC, Schwartz GG, Nicholls SJ, Ryden L, Neal B (2014). Effect of aleglitazar on cardiovascular outcomes after acute coronary syndrome in patients with type 2 diabetes mellitus: the AleCardio randomized clinical trial. JAMA.

[6] Pfeffer MA, Claggett B, Diaz R, Dickstein K, Gerstein HC, Kober LV (2015). Lixisenatide in patients with type 2 diabetes and acute coronary syndrome. N Engl J Med.

[7] Ray KK, Nicholls SJ, Buhr KA, Ginsberg HN, Johansson JO, Kalantar-Zadeh K (2020). Effect of apabetalone added to standard therapy on major adverse cardiovascular events in patients with recent acute coronary syndrome and type 2 diabetes: a randomized clinical trial. JAMA.

[8] Chamaria S, Bhatheja S, Vengrenyuk Y, Sweeny J, Choudhury H, Barman N (2018). Prognostic relation between severity of diabetes mellitus (on or off insulin) +/− chronic kidney disease with cardiovascular risk after percutaneous coronary intervention. Am J Cardiol.

[9] Dangas GD, Farkouh ME, Sleeper LA, Yang M, Schoos MM, Macaya C (2014). Long-term outcome of PCI versus CABG in insulin and non-insulin-treated diabetic patients: results from the FREEDOM trial. J Am Coll Cardiol.

[10] Gamble JM, Simpson SH, Eurich DT, Majumdar SR, Johnson JA (2010). Insulin use and increased risk of mortality in type 2 diabetes: a cohort study. Diabetes Obes Metab.

[11] Mendez CE, Walker RJ, Eiler CR, Mishriky BM, Egede LE (2019). Insulin therapy in patients with type 2 diabetes and high insulin resistance is associated with increased risk of complications and mortality. Postgrad Med.

[12] White WB, Cannon CP, Heller SR, Nissen SE, Bergenstal RM, Bakris GL (2013). Alogliptin after acute coronary syndrome in patients with type 2 diabetes. N Engl J Med.

[13] Borck PC, Guo LW, Plutzky J (2020). BET epigenetic reader proteins in cardiovascular transcriptional programs. Circ Res.

[14] Gilham D, Tsujikawa LM, Sarsons CD, Halliday C, Wasiak S, Stotz SC (2019). Apabetalone downregulates factors and pathways associated with vascular calcification. Atherosclerosis.

[15] Picaud S, Wells C, Felletar I, Brotherton D, Martin S, Savitsky P (2013). RVX-208, an inhibitor of BET transcriptional regulators with selectivity for the second bromodomain. Proc Natl Acad Sci USA.

[16] Bentzon JF, Otsuka F, Virmani R, Falk E (2014). Mechanisms of plaque formation and rupture. Circ Res.

[17] Nicholls SJ, Ray KK, Johansson JO, Gordon A, Sweeney M, Halliday C (2018). Selective BET protein inhibition with apabetalone and cardiovascular events: a pooled analysis of trials in patients with coronary artery disease. Am J Cardiovasc Drugs.

[18] Ray KK, Nicholls SJ, Ginsberg HD, Johansson JO, Kalantar-Zadeh K, Kulikowski E (2019). Effect of selective BET protein inhibitor apabetalone on cardiovascular outcomes in patients with acute coronary syndrome and diabetes: rationale, design, and baseline characteristics of the BETonMACE trial. Am Heart J.

[19] Gail M, Simon R (1985). Testing for qualitative interactions between treatment effects and patient subsets. Biometrics.

[20] Nicholls SJ, Schwartz GG, Buhr KA, Ginsberg HN, Johansson JO, Kalantar-Zadeh K (2021). Apabetalone and hospitalization for heart failure in patients following an acute coronary syndrome: a prespecified analysis of the BETonMACE study. Cardiovasc Diabetol.

[21] Noh M, Kwon H, Jung CH, Kwon SU, Kim MS, Lee WJ (2017). Impact of diabetes duration and degree of carotid artery stenosis on major adverse cardiovascular events: a single-center, retrospective, observational cohort study. Cardiovasc Diabetol.

[22] Venuraju SM, Lahiri A, Jeevarethinam A, Cohen M, Darko D, Nair D (2019). Duration of type 2 diabetes mellitus and systolic blood pressure as determinants of severity of coronary stenosis and adverse events in an asymptomatic diabetic population: PROCEED study. Cardiovasc Diabetol.

[23] Zoungas S, Woodward M, Li Q, Cooper ME, Hamet P, Harrap S (2014). Impact of age, age at diagnosis and duration of diabetes on the risk of macrovascular and microvascular complications and death in type 2 diabetes. Diabetologia.

[24] International Hypoglycaemia Study G (2019). Hypoglycaemia, cardiovascular disease, and mortality in diabetes: epidemiology, pathogenesis, and management. Lancet Diabetes Endocrinol.

[25] Nystrom T, Bodegard J, Nathanson D, Thuresson M, Norhammar A, Eriksson JW (2017). Second line initiation of insulin compared with DPP-4 inhibitors after metformin monotherapy is associated with increased risk of all-cause mortality, cardiovascular events, and severe hypoglycemia. Diabetes Res Clin Pract.

[26] Zhuang XD, He X, Yang DY, Guo Y, He JG, Xiao HP (2018). Comparative cardiovascular outcomes in the era of novel anti-diabetic agents: a comprehensive network meta-analysis of 166,371 participants from 170 randomized controlled trials. Cardiovasc Diabetol.

[27] Draznin B (2010). Mitogenic action of insulin: friend, foe or ‘frenemy’?. Diabetologia.

[28] Nicholls SJ, Tuzcu EM, Kalidindi S, Wolski K, Moon KW, Sipahi I (2008). Effect of diabetes on progression of coronary atherosclerosis and arterial remodeling: a pooled analysis of 5 intravascular ultrasound trials. J Am Coll Cardiol.

[29] Chaudhuri A, Rosenstock J, DiGenio A, Meneghini L, Hollander P, McGill JB (2012). Comparing the effects of insulin glargine and thiazolidinediones on plasma lipids in type 2 diabetes: a patient-level pooled analysis. Diabetes Metab Res Rev.

[30] Pilz S, Scharnagl H, Tiran B, Wellnitz B, Seelhorst U, Boehm BO (2007). Elevated plasma free fatty acids predict sudden cardiac death: a 6.85-year follow-up of 3315 patients after coronary angiography. Eur Heart J.

[31] Sarafidis PA, Bakris GL (2007). Non-esterified fatty acids and blood pressure elevation: a mechanism for hypertension in subjects with obesity/insulin resistance?. J Hum Hypertens.

[32] Malmberg K, Ryden L, Wedel H, Birkeland K, Bootsma A, Dickstein K (2005). Intense metabolic control by means of insulin in patients with diabetes mellitus and acute myocardial infarction (DIGAMI 2): effects on mortality and morbidity. Eur Heart J.

[33] Mellbin LG, Malmberg K, Norhammar A, Wedel H, Ryden L, Investigators D (2011). Prognostic implications of glucose-lowering treatment in patients with acute myocardial infarction and diabetes: experiences from an extended follow-up of the diabetes mellitus insulin–glucose infusion in acute myocardial infarction (DIGAMI) 2 study. Diabetologia.

[34] Sasso FC, Rinaldi L, Lascar N, Marrone A, Pafundi PC, Adinolfi LE (2018). Role of tight glycemic control during acute coronary syndrome on cv outcome in type 2 diabetes. J Diabetes Res.

